# Supplementary material for: White Matter Geometry Confounds Diffusion Tensor Imaging Along Perivascular Space (DTI‐ALPS) Measures
Source: Hum Brain Mapp. 2025 Jul 7;46(10):e70282. doi: 10.1002/hbm.70282 (PMC12231058; doi:10.1002/hbm.70282)
Supplement: Supplementary file 1 — Data S1. Supporting Information. [file HBM-46-e70282-s001.docx]

Supplementary material for:

White matter geometry confounds Diffusion Tensor Imaging Along Perivascular Space (DTI-ALPS) measures

Kurt G Schilling^1,2^, Allen Newton^1,2^, Chantal Tax^4,5^, Markus Nilsson^6^, Maxime Chamberland^7^, Adam Anderson^1,8^, Bennett Landman^1,2,8,9^, Maxime Descoteaux^3^

1. Vanderbilt University Institute of Imaging Science, Vanderbilt University Medical Center,

Nashville, TN, United States

2. Department of Radiology and Radiological Sciences, Vanderbilt University Medical Center,

Nashville, TN, United States

3. Sherbrooke Connectivity Imaging Lab (SCIL), Computer Science department, Université de Sherbrooke, Sherbrooke, Qc, Canada

4. Image Sciences Institute, University Medical Center Utrecht, Utrecht, The Netherlands

5. Cardiff University Brain Research Imaging Centre (CUBRIC), School of Physics and Astronomy, Cardiff University, Cardiff, UK

6. Department of Clinical Sciences Lund, Lund University, Lund, Sweden

7. Department of Mathematics and Computer Science, Eindhoven University of Technology, Eindhoven, The Netherlands

8. Department of Biomedical Engineering, Vanderbilt University, Nashville, TN, United States

9. Department of Electrical Engineering and Computer Engineering, Vanderbilt University, Nashville, TN, United States

**Abstract**

Introduction: The perivascular space (PVS) is integral to glymphatic function, facilitating fluid exchange and waste clearance in the brain. Diffusion Tensor Imaging Along the Perivascular Space (DTI-ALPS) has been proposed as a non-invasive marker of perivascular diffusion, yet its specificity remains unclear. ALPS measures assume that radial asymmetry in white matter diffusivity predominantly reflects PVS contributions. However, anatomical and microstructural confounds may influence these metrics.

Methods: We systematically evaluated potential biases in ALPS-derived measures using high-resolution, multi-shell diffusion MRI from the Human Connectome Project (HCP) and high-field imaging. Specifically, we examined (1) the prevalence of radial asymmetry across white matter, (2) the influence of crossing fibers on ALPS indices, (3) the impact of axonal undulations and dispersion, and (4) the spatial alignment of vasculature with white matter in ALPS-associated regions.

Results: Radial asymmetry is widespread across white matter and persists even at high b-values, suggesting a dominant contribution from axonal geometry rather than faster PVS-specific diffusion. Crossing fibers significantly inflate ALPS indices, with greater radial asymmetry observed in regions with a greater prevalence of crossing fibers. Furthermore, anisotropic axonal dispersion and undulations introduce systematic asymmetry independent of perivascular diffusion. Finally, high-resolution vascular imaging reveals substantial heterogeneity in medullary vein orientation, challenging the assumption that PVS consistently aligns with the left-right axis in ALPS regions.

Conclusion: ALPS indices are significantly influenced by white matter microstructure, including fiber crossings, undulations, and dispersion. These findings suggest that ALPS-derived metrics may not provide a direct measure of glymphatic function but rather reflect underlying axonal geometry. Interpretations of ALPS-derived metrics as biomarkers of glymphatic function must consider these anatomical complexities, and future studies should integrate advanced modeling approaches to disentangle perivascular contributions from white matter structure.

Keywords: ALPS; crossing fibers; DTI-ALPS; Undulation; Dispersion; Glymphatic System


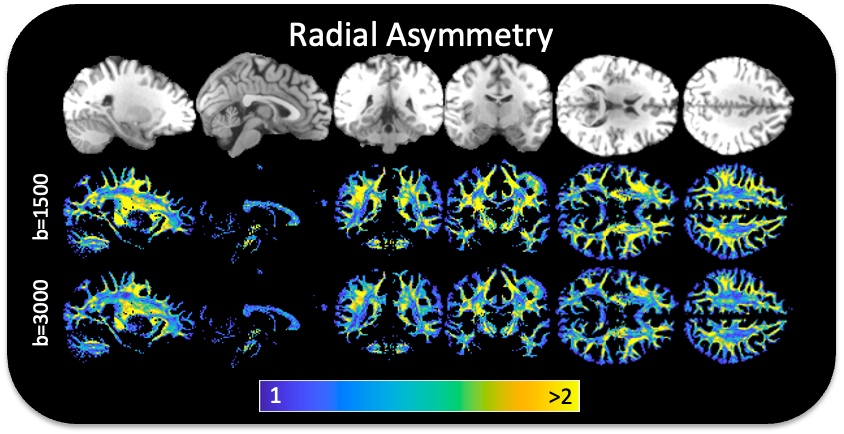


**Supplementary Figure 1**. (In parallel to main manuscript Figure 1 – Supplementary Figure 1 based on HCP-Aging data, main figure 1 based on HCP Young Adult Data). Radial asymmetry is widespread throughout white matter. Sagittal, coronal, and axial slices of an example HCP subject show radial asymmetry at all diffusion weightings, and throughout white matter, with most regions exhibiting average asymmetry values ~1.3-1.8, with many voxels >2.


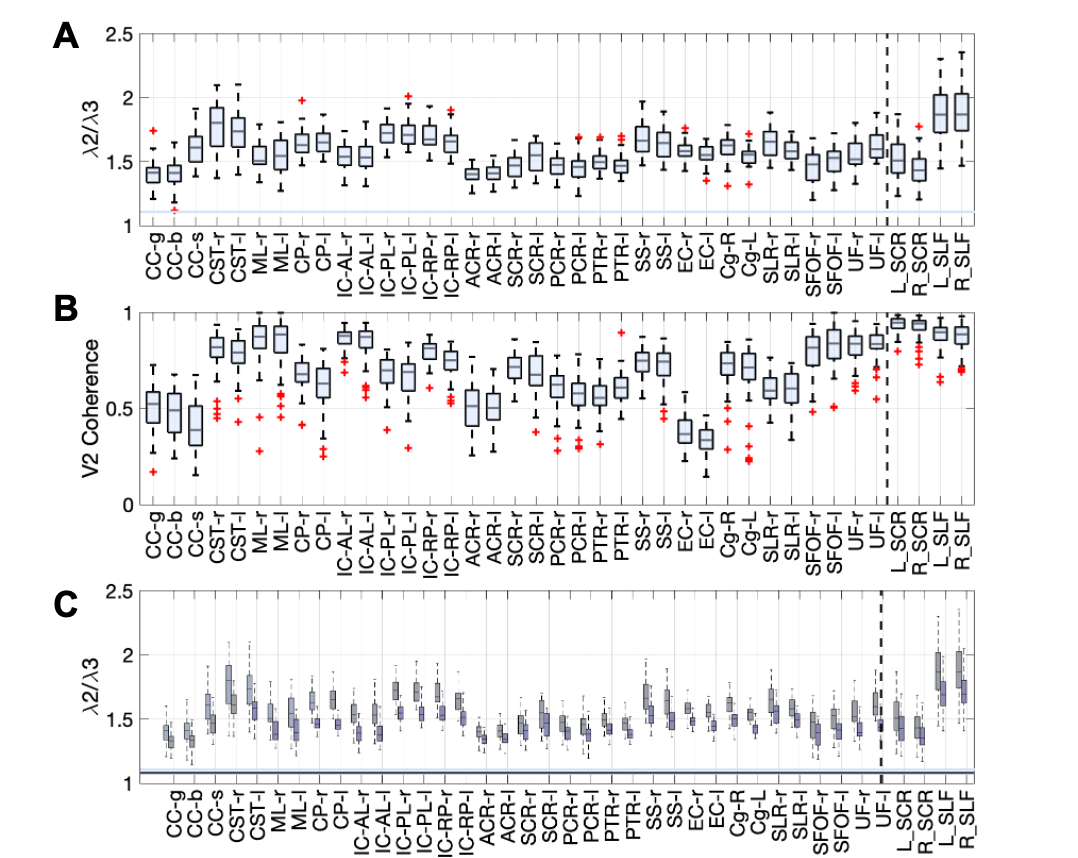


**Supplementary Figure 2**. (In parallel to main manuscript Figure 2 – Supplementary Figure 2 based on HCP-Aging data, main figure 3 based on HCP Young Adult Data). Most white matter regions exhibit radial asymmetry (“λ2/ λ3”). (A) Radial asymmetry (λ2/ λ3) is greater than 1, and greater than noise (color bar) for all JHU white matter and ALPS-specific regions (top, b=1000 data)(note that the four ALPS-specific regions are the rightmost regions in each plot to the right of the dashed vertical line). (B) The secondary eigenvector is coherent through these white matter regions (middle; V2 coherence), suggesting this is not a noise-related effect. (C) Radial asymmetry remains at all b-values, where b-values of 1000, 2000, 3000 s/mm2 are shown from light to dark for each region (bottom).


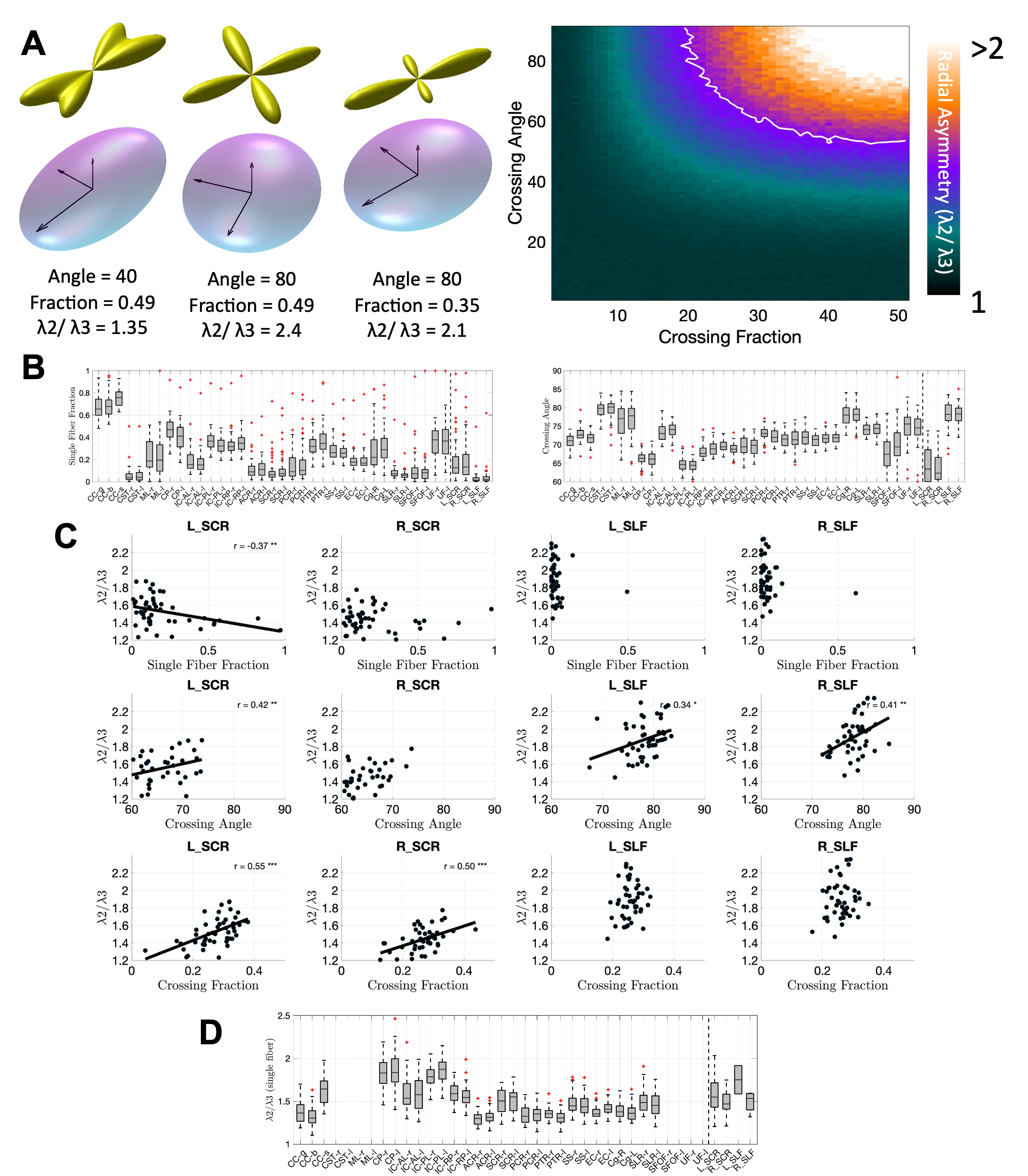


**Supplementary Figure 3.** (In parallel to main manuscript Figure 3 – Supplementary Figure 3 based on HCP-Aging data, main figure 3 based on HCP Young Adult Data). Most white matter regions contain crossing fibers, which contribute to radial asymmetry. (A) crossing fibers at different crossing angles and fiber fractions, resulting in radial asymmetry are visualized, as well as simulation results showing that increasing crossing angle and crossing fraction result in increased radial asymmetry. (B) Single Fiber Fraction and Crossing Angle are shown averaged in each white matter region. (C) Radial asymmetry is plotted against Single Fiber Fraction, Crossing Angle, and Crossing Fraction for ALPS-specific ROIs, across subjects, confirming that crossing fibers significantly influence asymmetry measures. Statistically significant correlations are shown as solid lines and indicated with asterisks (*p<0.05, **p<.01, ***p<.001). (D) Axial asymmetry remains even in single fiber voxels within each region.


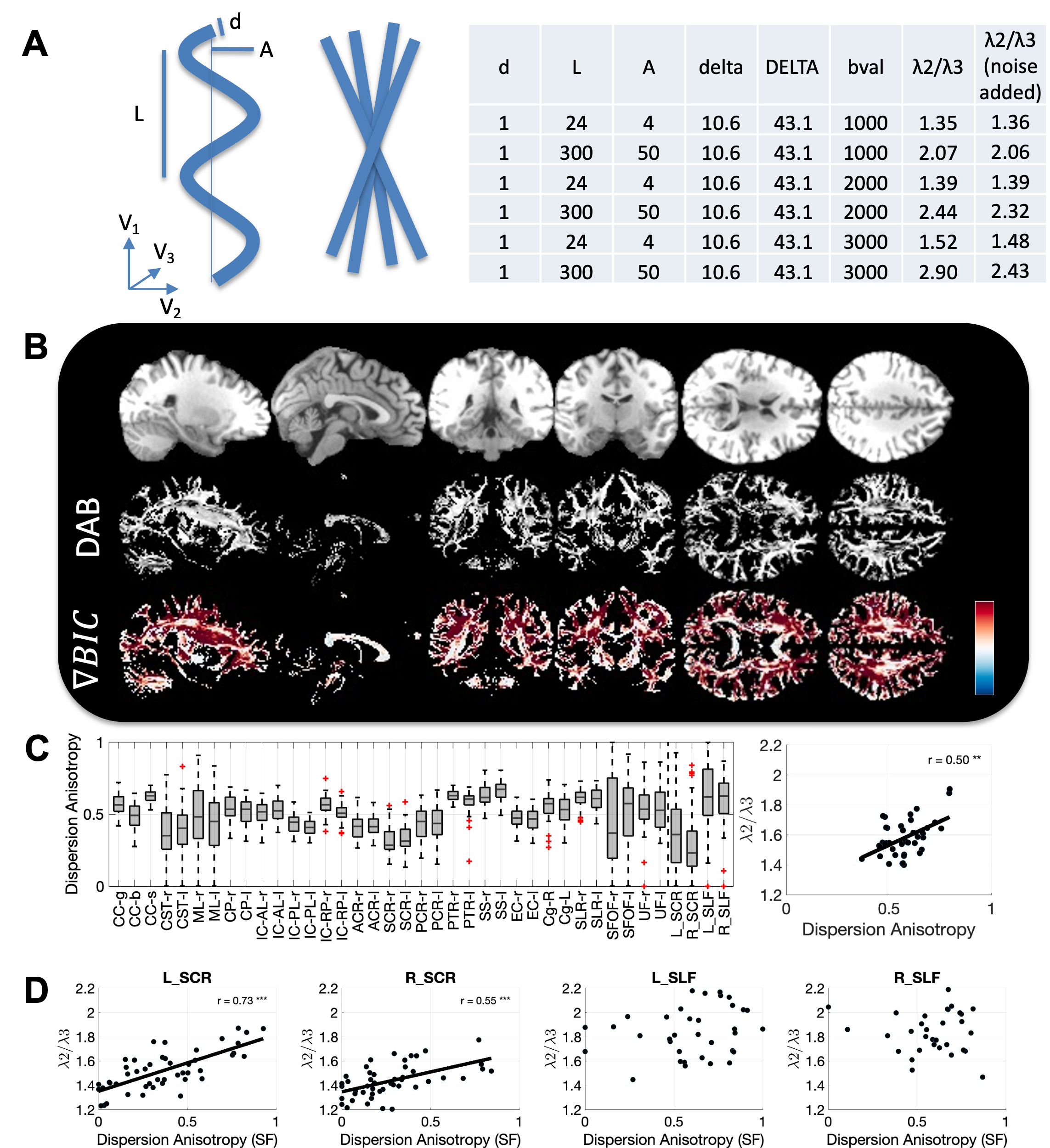


**Supplementary Figure 4.** (Parallel to Figure 5 of the main text – where Supplementary Figure 4 shows results for HCPA, main figure 5 shows results for HCP Young Adult data). Dispersion and Undulation contribute to radial asymmetry. (A) example undulation and dispersion cartoons, highlighting within-plane orientation dispersion. Simulation results with two substrates show that undulations can contribute to radial asymmetry on par with that observed in empirical data. (B) Example sagittal, coronal, and axial slices showing the Dispersion Anisotropy Index (DAB) (gray-scale) and the difference in BIC between Watson and Bingham distributions (Red indicates preference for Bingham, Blue indicates preference for Watson distribution). (C) Dispersion Anisotropy (DAB) across all white matter regions; radial asymmetry plotted against Dispersion Anisotropy (DAB) across all regions shows strong relationship between these two measures of asymmetry. (D). Similarly, Radial Asymmetry plotted against DAB across subjects shows strong relationships between these two measures in ALPS-specific ROIs, with a trend-line shown for regions with statistically significant associations (*p<0.05, **p<.01, ***p<.001).


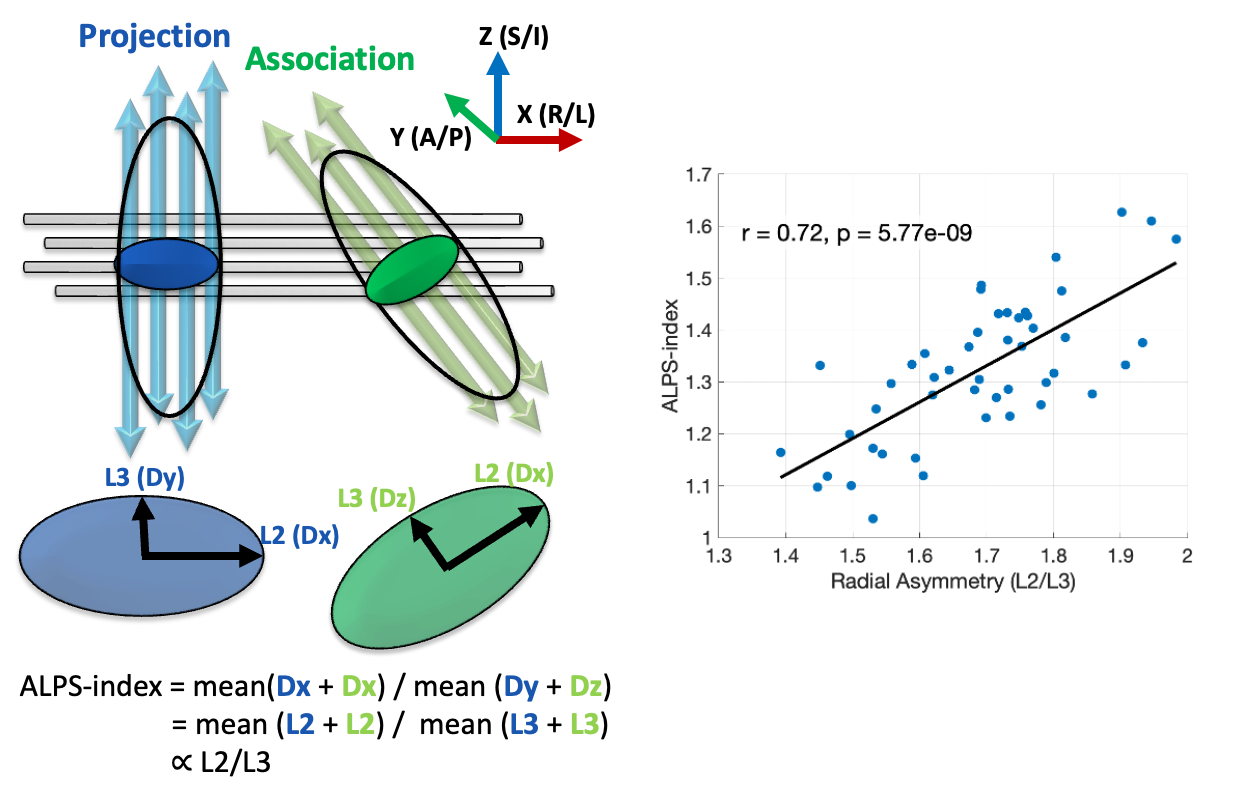


**Supplementary Figure 5.** (Parallel to Figure 8 of the main text – where Supplementary Figure 5 shows results for HCPA, main figure 8 shows results for HCP Young Adult data). The ALPS-index is a measure of radial diffusivity asymmetry. Left: Schematic depiction of the anatomical configuration underlying the ALPS method. Medullary veins (gray cylinders) run predominantly in the right–left (x) direction, orthogonal to dominant fiber orientations of projection (blue, superior–inferior) and association (green, anterior–posterior) tracts. In these regions, diffusion tensor eigenvectors align such that λ₂ corresponds to the x-direction (Dx), and λ₃ reflects diffusivity orthogonal to both the fiber axis and perivascular direction (Dy in projection fibers, Dz in association fibers). The ALPS-index is computed as the mean of Dx across projection and association regions divided by the mean of Dy and Dz - effectively λ₂/λ₃. Right: Empirical relationship between radial asymmetry (λ₂/λ₃) and the ALPS-index across regions of interest (calculated using the automated methods described in [40]). A strong, statistically significant, positive association (r=0.56 for HCP, r=0.72 for HCP-A, supplementary material) confirms that the ALPS-index is fundamentally a measure of radial asymmetry. Thus, any feature that alters radial asymmetry (investigated throughout the study), influences the ALPS index.


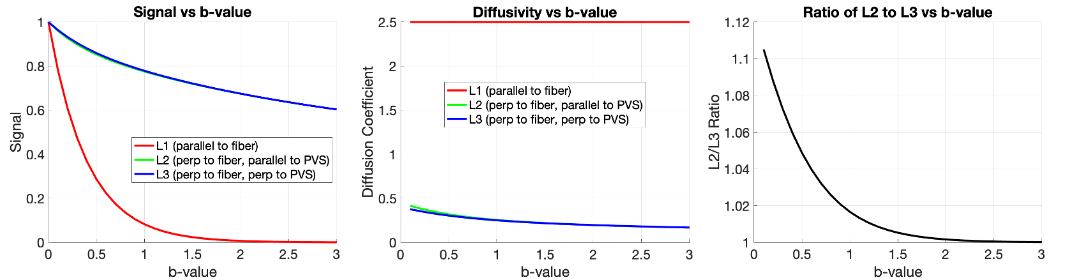


**Supplementary Experiment 1.** Investigating how a true PVS/APLS effect influences our metrics of asymmetry, i.e., would a true ALPS-related changes influence the asymmetry index? A simple 3 compartment model was simulated, incorporating an asymmetric PVS compartment with a volume fraction of 10% and diffusivities consistent with prior experimental estimates [53] (along perivascular space diffusivity=3, radial to perivascular space diffusivity=2.5), with an intra-axonal compartment (axial diffusivity = 2.5, radial diffusivity = 0), and an extracellular compartment (isotropic diffusivity = 2.5). (Left) The signal was simulated across a range of b-values for (1) along the WM fiber and perpendicular to PVS (λ1) (2) perpendicular to the fiber and parallel to PVS (λ2), and (3) perpendicular to the fiber and perpendicular to PVS (λ3):

S1 = 0.1*exp(-b*2.5) + 0.4*exp(-b*2.5) + 0.5*exp(-b*2.5);

S2 = 0.1*exp(-b*3)   + 0.4*exp(-b*0)   + 0.5*exp(-b*0.3);

S3 = 0.1*exp(-b*2.5) + 0.4*exp(-b*0)   + 0.5*exp(-b*0.3);

From this, the diffusivities (Middle) are calculated along each direction, and the ratio of λ2 to λ3 (i.e., the asymmetry index) was derived (Right). With these assumed diffusivities, high b-values are not sensitive to asymmetric PVS effects.
